# Acceptability and feasibility of online delivery of chair-based yoga for older adults with multimorbidity – lessons from a process evaluation of the gentle years yoga trial

**DOI:** 10.1186/s12906-025-04838-6

**Published:** 2025-03-17

**Authors:** Lesley Ward, Laura Bissell, Jenny Howsam, Garry A. Tew, Laura Wiley, Fiona Rose, Camila Sofía, Maturana Palacios, Tim Rapley

**Affiliations:** 1 Department of Social Work, Education and Community Wellbeing, Northumbria University, Newcastle upon Tyne, UK; 2 British Wheel of Yoga Qualifications, Sleaford, UK; 3 Institute for Health and Care Improvement, York St John University, York, UK; 4 York Trials Unit, Department of Health Sciences, University of York, York, UK

## Abstract

**Background:**

Yoga is a safe, effective, and popular practice among older adults, and amenable to online delivery. The Gentle Years Yoga randomised controlled trial compared the impact of a chair-based yoga programme to usual care on the health-related quality of life of older adults with multimorbidity. This embedded, longitudinal process evaluation qualitatively explored experiences and acceptability of online delivery of the trial intervention.

**Methods:**

A subset of trial participants randomised to receive the 12-week online yoga programme, together with the trial yoga teachers, were purposively recruited to semi-structured interviews. Individual interviews were conducted via Zoom or telephone, audio-recorded, independently transcribed, and thematically analysed. Online observations were conducted of one class delivered by each teacher.

**Results:**

Eighteen yoga participants (66–91 years; 2–8 chronic health conditions) and nine teachers were interviewed once (*N* = 12) or twice (*N* = 15) from October 2020 to April 2022. Five themes predominated, common to both groups. (1) Accessibility. Reduced communication and engagement inherent to online delivery were mostly outweighed by its removal of access barriers and provision of anonymity and distraction-free environment. (2) Technology issues. While digital literacy was variable and a barrier for some, simplified access procedures and basic audiovisual instruction optimised class engagement. (3) Delivery adaptations. Key facilitation techniques included simple, repetitive instructions, increased demonstration, personalised communication, and visibility-enhancing clothing. (4) Safety. Concerns were minimal, and mostly related to restricted visual and positional information inherent to face-to-face classes. (5) Implications and implementations. Online delivery was considered viable and potentially appealing for anyone experiencing issues accessing face-to-face classes outside the home. Potential solutions to online attendance barriers included equipment loan schemes and digital learning courses using existing community-based infrastructures.

**Conclusions:**

Online chair-based yoga classes were feasible and acceptable to participants and teachers, and preferrable to face-to-face delivery by some. IT issues were minimal, and mainly resolvable through simple access processes and educational information. Accessibility advantages suggest online yoga may be suitable for a broad demographic, independent of age or health status. Establishing connections with existing health and community-based organisations presents a potential pathway for developing an equipment loan scheme to improve accessibility for those with financial access barriers.

**Trial registration:**

ISRCTN ISRCTN13567538. Registered 18 March 2019.

**Supplementary Information:**

The online version contains supplementary material available at 10.1186/s12906-025-04838-6.

## Introduction

The world’s population is ageing, with older adults (65 years and over) estimated at almost a quarter of the UK population by the 2040s [1]. Concomitant with an ageing population is increased disease burden. Multimorbidity, the presence of two or more long term health conditions (LTHCs), is common in older adults [2] and carries an economic burden of increased health resource and pharmaceutical usage [3]. However, many health conditions common to older adults have associated health behaviour risk factors, presenting a pathway for non-pharmaceutical self-management.

Physical inactivity is a risk factor for many LTHCs including osteoarthritis, heart disease, and type 2 diabetes [4]. Conversely, research suggests physical activity and planned therapeutic exercise appear safe and beneficial for both older and multimorbid adults [4, 5], are associated with independent living and improved biopsychosocial outcomes [6, 7], and may reduce the risk of serious adverse events associated with multimorbidity [4]. Health guidelines suggest discussing options such as exercise or mind-body practices for the non-pharmaceutical management of multimorbidity and LTHCs [8–11]. However, the prevalence of older adults meeting recommended physical activity guidelines is low [7] and may be associated with health-related issues including restricted mobility, upper limb dysfunction, and depression [5].

Yoga, a mind-body practice typically comprising physical, breathing, and relaxation techniques, is an increasingly popular and health-inclusive physical activity among older adults [12]. Acceptable, feasible, and effective for improving physical and psychological outcomes in older adults [13, 14], yoga is also associated with symptom improvement in a range of age-associated LTHCs (e.g [15–21]).

Telehealth, the delivery of health-based education via telecommunication techniques, has become an increasingly important health service pathway in the context of the Covid-19 pandemic [22]. Evidence to date suggests yoga is amendable to this delivery format. Online yoga (tele-yoga) has been deemed safe, acceptable, and beneficial to physical and mental health for several clinical conditions prevalent in older adults, including cancer, chronic obstructive pulmonary disease, and heart failure [23–25].

Telehealth is also suitable for older adults. As the fastest growing group of internet users [26], older adults’ internet use is associated with improved mental health and self-management competency and decreased social isolation [27]. However, an estimated 30% of UK older adults experience digital exclusion [27], commonly associated with financial, health, and digital literacy barriers [27, 28]. Compounding these issues is an ageist stereotype of technological inability among older adults, knowledge of which negatively impacts their use of technology [29]. Older non-users of the internet cite perceptions rather than direct experiences as reasons for non-use, perceiving the internet as too complicated, difficult to learn, of little use, and having potential security issues [28, 30, 31].

### The Gentle Years Yoga trial

The Gentle Years Yoga (GYY) trial was a multi-site randomised controlled trial with embedded economic and process evaluation, investigating the effectiveness of a chair-based yoga programme compared to usual care for older adults (65 + years) with multiple LTHCs. Full trial details and quantitative results are reported separately [32–34]. Briefly, the 12-week programme delivered once-weekly, 75-minute group classes, primarily comprising physical, breathing, and relaxation practices, with optional 15-minute social time afterwards. Delivery occurred across two pilot and two main trial phases, at sites in England and Wales.

The programme was developed for face-to-face delivery by yoga teachers trained in GYY. However, Covid-19 social restrictions instigated prior to the second pilot phase of the trial necessitated a rapid and unplanned transition to online delivery via Zoom videoconferencing [35] transforming the GYY programme to a tele-yoga format [36]. Additional recruitment criteria excluded those without internet access or the ability to use it, or a suitable device to view the online classes.

The GYY programme content remained unchanged for online delivery; however, a one-to-one Zoom session occurred prior to the first group class, enabling teachers to assist participants with Zoom access, room set-up, and sharing of health and safety information. Online classes remained the sole delivery option in the first main trial phase; both online and face-to-face classes were delivered in the final trial phase as Covid-19 social restrictions lifted.

### Aims

To effectively advance and optimise the field of tele-yoga, more information is required from both recipients and yoga teachers into facilitating this experience [37]. The aim of this current study was to undertake an in-depth qualitative exploration of participant and yoga teacher experiences and feasibility of the online delivery of the GYY programme. This study is reported with reference to guidelines for qualitative research [38].

## Method

### Study design

This qualitative study formed part of the process evaluation of the GYY trial and comprised one-to-one interviews with trial intervention participants and yoga teachers, and observations of class delivery. All participants and yoga teachers taking part in the observations and interviews provided written informed consent.

### Recruitment

Recruitment occurred across the second pilot phase and both main phases of the trial, following the introduction of online delivery of the 12-week yoga programme. Trial participants (henceforth referred to as participants) who were both allocated to receive the online yoga programme and had indicated interest in the process evaluation interviews on their trial consent form were eligible to take part; participants receiving face-to-face delivery of the yoga programme were not eligible. Participant information sheets and consent forms specific to the interviews were provided. Participant sample size was not pre-determined; recruitment continued until meaning saturation occurred [39]. The interview recruitment process was replicated with all nine yoga teachers (henceforth referred to as teachers) delivering the online GYY programme.

Due to the high level of interest in the interviews among yoga participants, and in line with qualitative methodology [40], a purposive sampling strategy was undertaken by the process evaluation team (LW; TR) to ensure selected participants represented the demographic breadth of age, gender, ethnicity, and index of multiple deprivation score (a UK indicator of relative deprivation ranging from 1 to 10, with 1 indicating the most deprived area), study sites, and the number, severity, and type of physical and mental health LTHCs. To minimise positive response bias related to intervention attendance, participants were eligible for the interviews regardless of their level of class attendance. Participants were contacted by the qualitative researcher (LW) via telephone or email to discuss any questions. Once written informed consent was gained an interview date and format (Zoom or telephone) was determined.

Following initial interviews, a representative subset of participants was invited to take part in a second interview to explore any longer-term impacts of their online yoga experience; as per initial interviews, recruitment continued until meaning saturation was reached [39]. Recruitment to the observations followed a similar process; declining to take part in the observations or interviews did not impact a participant’s trial status.

### Data collection

Interview and observation data were collected from October 2020 to April 2022. Observations occurred at various timepoints across the 12-week yoga intervention; likewise, initial and follow-up interviews were conducted at various timepoints during and after the 12-week intervention. This breadth of data collection timepoints was used to maximise the breadth of participant and teacher experiences of the online programme across time.

All interviews and class observations were conducted by an experienced qualitative researcher (LW); field notes supplemented formal data collection and were regularly reviewed by the process evaluation team (LW; TR), enabling reflection and identification of any potential assumptions or biases that may have occurred in the collection process.

One-to-one interviews were conducted via Zoom or telephone, as per individual choice. Interview questions were based on a semi-structured interview schedule, developed specifically for this study (Supplementary file 1). The interview schedule was iteratively adapted by the process evaluation team (TR, LW) in line with new, unanticipated, and emerging data; longitudinal interview schedules were additionally tailored to explore changes over time. Interviews explored general biopsychosocial health and activity, IT competency, pragmatic and experiential aspects of online delivery, and intentions for sustainability of online yoga post-trial. Where possible, wording of core questions was adapted to both participants and yoga teachers, to enable comparison of data from the aspect of different stakeholders. To supplement the interview data, seven online class observations were conducted, with field notes written in real time to minimise recall bias. Observations were conducted prior to a teacher’s interview where possible, to inform and tailor questions relating to online class delivery.

### Data analysis

Interviews were audio-recorded, independently transcribed verbatim, and checked by the process evaluation researchers (LW; TR) against the original audio file for accuracy. Both interview transcripts and observation field notes were anonymised to remove information identifying any individuals or delivery sites. Data analysis by the process evaluation researchers was iterative throughout the trial, conducted according to the standard procedures of rigorous qualitative [40], longitudinal [41], and thematic [42] analysis. Analytic stages included open and focused coding, constant comparison, deviant case analysis, and within- and across-participant thematic charting to explore both single point and longitudinal experiences [43, 44]. Interim findings were presented at trial management meetings, to enable wider trial staff to input into the interview schedules and preliminary findings.

## Results

### Demographics

Eighteen yoga participants from seven study sites were interviewed once (*N* = 6) or twice (*N* = 12) across the pilot (*N* = 10) and main (*N* = 8) trial phases (October 2020 to April 2022), providing a 30-interview data set. Two participants who were invited for a follow-up interview after their initial interview did not provide data; one declined the second interview, and the other was unable to be contacted.

Individual interviews were conducted across a range of different time points, from Week 4 of the 12-week intervention to 8 months post-intervention; interview duration ranged from 24 to 98 min. Participant demographics represented the breadth of the wider trial cohort: 56% female (*N* = 10), aged 66–91 years (mean age 72 years), predominantly White British (*N* = 16, 89%), with index of multiple deprivation scores ranging from 1 to 10. Twelve participants lived with others and six lived alone. Number of LTHCs per participant ranged from two to eight (mean 4.9); most common of the 27 self-reported health issues were osteoarthritis (*N* = 10, 56%), hypertension (*N* = 9, 50%), pulmonary issues (*N* = 7, 39%), and anxiety (*N* = 6, 33%).

All nine yoga teachers delivering the online classes were interviewed either once (*N* = 6) or twice (*N* = 3), providing a 12-interview data set. Interview timepoints ranged from week 4 of the 12-week programme to 3 weeks post-intervention, and interview duration ranged from 45 to 90 min. Supplementary observations of seven online classes were conducted in the pilot and main phases, across the early (week 4), middle (week 7), and late (week 11) intervention phases. The teachers were predominantly female (89%), with yoga teaching experience ranging from 4 to 35 years, and GYY teaching experience ranging from newly qualified to 5 years. Three of the nine teachers had delivered the GYY programme in both a face-to-face and online format during the pilot phases of the trial, and all teachers had experience teaching community-based yoga classes prior to the GYY trial.

### Thematic outcomes

Teachers and participants shared similar, overlapping views of the online yoga classes. Five themes predominated across both the initial and longitudinal data collection periods, covering accessibility, technology issues, adaptations, and safety, and suggestions for future implementation of GYY classes. Supporting verbatim quotes are provided below. To maintain participant and yoga teacher anonymity, quotes are provided with an identification (ID) number only, numbered sequentially as they appear in the text (e.g. *ID1).*

### Accessibility

#### Removal of attendance barriers

An overarching theme supporting online delivery was inclusion via accessibility. Several barriers to online classes were noted by teachers and participants, including lack of suitable computer equipment or a quiet space to practice. However, these were outweighed by the physical and temporal benefits of online classes. Notably, these benefits were independent of Covid-19 social restrictions, and thus directly contributed to participants noting a preference and/or intention to continue with online classes once Covid-19 social restrictions were lifted.

Integral to accessibility was the removal of travel to onsite classes. This improved class access for those with transportation issues related to mobility, acute health issues, and adverse weather, enabling consistent yoga practice independent of external or health-related factors:

*‘It’s easier with it being on Zoom*,* because I don’t drive anymore. I used to*,* but because of the arthritis in my neck and my knees*,* I was finding it quite painful.’ Interview participant ID1*.

Online delivery also removed geographical attendance barriers for those living in areas where face-to-face classes or public transport were unavailable. Furthermore, online classes expanded participants’ choices post-trial; with several participants choosing to continue their practice with their trial teacher or explore non-GYY online options rather than attend local face-to-face classes:

*‘I’ve signed up for another 10 classes with [trial yoga teacher]*,* because*,* it was a time that was convenient*,* and having done the original 12 classes*,* although we’d never actually physically met*,* I did feel as though we knew each other*,* to some extent.’ Interview participant ID2*.

Finally, online delivery provided temporal accessibility. Many participants cited time restrictions due to a busy lifestyle, work, or carer responsibility, and valued the convenience of their yoga practice only involving the time commitment of the actual class in a space of their choice:

*‘…the fact that it was on Zoom worked very well because it wasn’t so time-consuming in my day. It was just would be the hour and a quarter*,* whatever*,* not more than that*,* rather than having to drive and dedicate a whole morning or afternoon to it.’ Interview participant ID3*.

#### Enhanced learning environment

Unique to participants was the perception that online classes offered a level of focus and anonymity unavailable in a face-to-face situation. Audiovisual settings of muted microphones and speaker view enabled participants to see and view only the yoga teacher. While some participants missed the sociality of group interaction, others found this quiet environment, without the distraction of others, enhanced their learning experience:

*‘But with the Zoom*,* I’ve found I haven’t got anybody else to concentrate on besides [yoga teacher] and myself. There’s no distraction. And that’s strange for me because I am a very sociable person*,* but I just think it’s really*,* really – For me*,* it’s really – you know*,* it keeps me focused.’ Interview participant ID4*.

Participants choosing speaker view felt a direct and exclusive connection with the teacher, within the identity of the wider group class. The ability to focus solely on the teacher without distraction in turn provided a level of focus, anonymity, and emotional security not available in face-to-face settings:

*‘[…] it cuts out that embarrassment factor*,* that you don’t want to make a fool of yourself with these particular moves*,* you know*,* and poses.’ Interview participant ID5*.

This environment of anonymity was most appreciated by those socially uncomfortable in group environments, those who did not like others seeing their visible mobility restrictions, and male participants uncomfortable at learning a new activity in front of a predominantly female class.

#### Restricted information access

Restrictions inherent to online delivery were noted regarding bidirectional visual, spatial, and verbal information. The two-dimensional nature of on-screen viewing, combined with often restricted full body views of participants due to space or camera limitations meant participants and teachers received limited visual information of each other compared to that gained in person:

*‘I think the one big thing is that the teacher*,* if you’re face-to-face and you’re not in the right position for example*,* and you need slight changes to your body positions to get the best out of the particular exercise*,* she can actually walk over to you and move your leg*,* move your arm. She can’t do that over Zoom. She can just make a comment and say*,* “You need to lift your leg up*,*” or that sort of thing.’ Interview participant ID6*.

However, for some participants these restrictions were mediated by receiving real-time positional feedback via screen view, which provided them with positional information for self-adjustment not available in face-to-face classes:

*‘And certainly*,* looking at myself on the screen*,* because I can see myself on the corner screen*,* and I think*,* sit up*,* you’re not doing it properly [laughter].’ Interview participant ID7*.

#### Restricted social engagement

Participant views regarding social restrictions inherent to online delivery were mixed. For some, the social aspect of the yoga classes held no interest, and they instead preferred the focussed environment offered by the Zoom classes. Conversely, participants who preferred a level of sociality in their activities felt this was lacking in the online classes. They missed both the physical presence of their peers for social support and interaction, and of the class teacher for providing personalised positional information and adaptations. While optional social time was offered at the end of classes, those of a quieter nature found it difficult to engage in this online environment:

*‘I’m not a ‘shouting over people’ person*,* you know*,* I’m not an attention-grabbing person. And I found it very hard to get a word in. You know*,* she’d say*,* “How are you?” And I’d get as far as saying*,* “Oh well*,* I’m not so bad*,*” and then somebody would be yabbing away.’ Interview participant ID8*.

#### Cost-benefit balance

Online yoga classes carried a cost of restricted audiovisual communication and social engagement compared to face-to-face activities. However, for most participants these were outweighed by the benefits of accessibility, both in relation to Covid-19 restrictions and to their general health and lifestyle. As such, even those who expressed a clear preference for face-to-face interactions appreciated being able to access an activity during Covid-19 restrictions.

Yoga teachers weighed the accessibility benefits of online delivery against the cost of time and, by association, finances. Both their trial-based and private online classes provided the opportunity to engage with a more physically and geographically diverse audience compared to face-to-face delivery, primarily due to the removal of mobility and transport barriers:

*‘You’re able to keep in touch with people who are not just in your locality*,* people who might want to come to your yoga class but they never could because it was too far to drive.’ Yoga teacher ID1*.

However, teachers noted that in their private online classes, these accessibility benefits were offset by increased expenses, primarily associated with the necessity of providing one-to-one appointments prior to online classes to discuss health and IT issues. From a self-employed perspective, time is money, and this additional time expense outweighed any savings associated with teachers not having to rent premises for face-to-face classes.

### Technology issues

Both participants and teachers experienced technical and skills-based issues related to general digital literacy and specific Zoom processes. Following evaluation of online delivery during the pilot phase of the trial, additional teacher training and a one-to-one session between teachers and participants were instigated for delivery during the main trial phase. While these measures improved access and audiovisual issues, new issues related to internet and Zoom connectivity provided challenges. As such, participants and teachers stated an ongoing need for access to IT support from trial staff across the 12-week programme period.

#### Digital literacy

Digital literacy was variable across both participants and yoga teachers, ranging from basic computer skills to highly competent workplace use. Most participants were active digital engagers, with in situ computer equipment and internet; some had to source an external camera for the Zoom classes. All participants were competent with email, most were familiar with basic search engines including Google or YouTube, and several used Skype and WhatsApp for social communication.

Some participants initially viewed their limited IT knowledge as a barrier to attending Zoom-based classes. However, these skill-based concerns were mostly resolved with basic training and support from family members and the teachers, with evidence of increased digital literacy over the 12-week intervention:

*‘I’ve got a bit more used to it*,* I was able to open [Zoom] up myself this morning without my wife here.’ Interview participant ID2*.

#### Optimising zoom access

Pre-trial Zoom use was variable. Some participants and teachers used Zoom pre-Covid-19, others were self-taught to maintain family, social, and work communications during Covid-19 restrictions, and others had no knowledge of it. Accordingly, simplicity of set-up and instruction was key to its access and use. As all participants were email-literate, embedding a clickable link into an email provided simple one-step access independent of digital literacy:

*' … [son] said*,* “Literally*,* go to that and click on it.” It was very simple*,* because I’m really a technophobe. I mean*,* I haven’t got a clue.’ Interview participant ID7*.

Conversely, additional steps requiring passwords and waiting rooms were challenging for those unfamiliar with video conferencing. This was compounded by some yoga teachers new to Zoom not knowing how to alter settings to remove these additional access steps. This extended process prevented some participants from timely access to classes, and was associated with disinterest in online yoga following trial completion:

*‘About two or three classes*,* I’ve got so frustrated that it wouldn’t link in and I couldn’t get on*,* so I missed at least 10 minutes*,* 15 minutes of some classes. Not because I hadn’t turned up or anything*,* it was just the fact that I couldn’t get on*,* it wasn’t accepting what I was putting in.’ Interview participant ID9*.

Participants were further challenged by audiovisual settings, including muting microphones and changing between gallery view during social time and speaker-only view during class instruction. Again, several teachers were unaware of how to adjust these settings for their participants, with their knowledge gap further compounded by unfamiliarity with the wide variety of participant devices:

*‘[…] I think that’s the first time I’ve actually come across someone working on a tablet which isn’t an iPad*,* which I don’t know*,* I don’t have such a device. I’ve got no idea of it.’* Yoga teacher ID2.

### Adaptations to optimise delivery

Participants and teachers noted several adaptations to mitigate the communication restrictions inherent to an online delivery format. These were primarily centred around basic digital literacy instruction, interpersonal communication, and teaching style.

#### Pre-class one-to-one session

Both teachers and participants supported the pre-class Zoom session instigated for online delivery. These sessions mediated participant concerns regarding the digital literacy required to access the Zoom-based classes, and, to the best of the teacher’s ability, provided participants with basic audio-visual instruction. Participants further noted that one-to-one meetings were common and/or required for some face-to-face community classes they attended such as Pilates, where the instructors used these sessions to gain knowledge of any individual health-related adaptations that may be required in the group classes.

Within the context of the GYY trial, the one-to-one sessions were instigated to address technology issues. However, both groups cited the primary benefit was establishing a personal connection and providing the opportunity for participants to privately share health information and address any concerns regarding yoga. This connection was particularly valued by participants who experienced anxiety in new social interactions, with the broader health picture enabling teachers to tailor their group class content around individual participant needs:

*‘I would have been going into a room full of strange people*,* totally*,* it would have been a lot more anxiety-provoking if I hadn’t at least known that the tutor was okay.’ Interview participant ID8*.

Additionally, the teachers noted several safety benefits. The one-to-one session enabled them to undertake a visual risk assessment of the participant’s practice space, mobility, and audiovisual requirements, and position their chair to optimise mutual visibility during the classes.

#### Adapted teaching style

Both participants and teachers viewed proactive teacher facilitation as key to the online classes. Teachers noted that their teaching adaptations were mainly based on mobility, cognitive, and audiovisual issues common to older adults and as such were independent of a face-to-face or online delivery format. Adaptations included slow, simple, repetitive instructions, wearing a microphone, and using verbal adjustments as a proxy for physical adjustments.

Verbal adaptation was supplemented with a proactive physical presence, offsetting restricted positional information inherent to single camera viewing angle and two-dimensional screens. Teachers increased their use of demonstration compared to face-to-face classes, demonstrated postures from multiple angles, moved closer to their screen to demonstrate smaller movements or breathing practices, and encouraged non-verbal communication such as hand and facial gestures to inform their subsequent teaching instruction:

*‘You use the camera. If you’ve got fine movements*,* like hand movements*,* I will come into the camera*,* you know*,* and show my fingers*,* or the mudra I’m doing*,* repeat it*,* so that people can see. So*,* knowing that if you are further back you need to come in to use the camera*,* to make sure people can see*,* that would be one.’ Yoga teacher ID1*.

Supplementing this proactive facilitation, participants favoured personalised communication over generic group feedback. Some teachers opposed using names, viewing it as singling people out. However, participants favoured this approach as confirming they were receiving personalised instruction within the context of a group class:

‘*Then [trial yoga teacher] might say*,* “[Name]*,* are you okay?” Or*,* “[Name]*,* are you okay? [Name]*,* are you okay?” or something like that. So*,* we’re all on mute*,* but it’s – And then [YT] will say*,* you know*,* “Thumbs up.” So*,* you do feel you’re part of a group*,* but not sort of seeing the group.*’ *Interview participant ID4*.

#### Optimising visibility

Optimising mutual visibility required compromise; participants needed to be close enough to their computer screens to see the teacher, yet far enough away for the teacher to see their positional information. Most teachers agreed that, from a safety perspective, the participants’ view of them had priority over their view of the participants. As such, screen closeness meant participants rarely presented a full body view in either seated or standing positions, with teachers relying on observation and nonverbal cues to determine their off-screen body positioning.

Camera quality further impacted participants’ view of the teacher, as many integrated computer cameras are programmed for close viewing. Class observations noted some teachers needed to position themselves a distance from the screen to present a full body view, making it difficult for participants to see their facial expressions and smaller movements. Where possible, teachers adapted to this by purchasing camera equipment with wide-angle views and advanced lighting features. However, some noted the caveat that the software needed to program advanced equipment could impact connectivity for those with weak internet signals, and was not often able to be used during teaching:

*‘If I use the [brand] software*,* which does all sorts of wonderful things and sorts your lighting out for you automatically*,* our internet is too slow. And if it’s been on a while you start to get a mismatch between what I’m doing and there’s a kind of a delay*,* there’s a time lag.’ Yoga teacher ID3*.

Teacher visibility was further influenced by their clothing. Monotone dark clothing restricted positional information from the teachers’ thighs to upper body in both a seated and standing position. Conversely, positional information was clear in those wearing a different light coloured top and leggings. Visibility was further enhanced when seated against a contrasting, monotone background.

### Safety

The yoga teachers had minimal safety concerns for online yoga beyond those inherent to face-to-face classes. This centred around their unanimous view that frequent safety reminders are integral to teaching yoga, regardless of the delivery format. They felt the safe delivery of the GYY trial classes was further enhanced by adaptations instigated by the trial team to facilitate participant visibility and communication. These included issuing the teachers with large TV monitors, providing them training on audiovisual computer settings, and reducing the size of the online classes compared to the face-to-face classes.

The teachers’ inability to gain head-to-toe views of all participants was compensated for by their confidence in both the content and instruction of their classes and their experience of reading non-verbal cues, such as facial expressions and upper body posture as a proxy for foot positioning and balance:

*‘I always make sure I can see their face and their upper part of their body at the very least*,* so I can see what they’re doing with their arms*,* I get that feedback as to whether they’ve got any limitations in their upper body.’’ Yoga teacher ID4*.

While generally in agreement with teachers, some participants noted the restricted visual and positional information inherent to online delivery may impact safety related to incorrect postural form:

*‘I felt the downside of that would be that*,* if she couldn’t correct me early enough in my process of learning to do the yoga*,* I would get stuck in a way of doing it that was incorrect. I don’t think it would probably do much damage*,* but it might do*,* and there’s that bit of doubt.’ Interview participant ID9*.

### Implications and implementations

While some participants looked forward to the sociality of rejoining their face-to-face activities, both teachers and participants considered online delivery as a viable option for the future. Many participants noted that online attendance had become the default option for many of their community-based activities during Covid-19 restrictions and would continue with this format once restrictions were lifted due to the accessibility and convenience benefits inherent to home-based engagement. Teachers mirrored these opinions of delivery format. While they had, or would be, resuming face-to-face classes in line with the lifting of Covid-19 restrictions, many indicated they would also continue to offer online classes in their private businesses due to customer demand:

*‘Well*,* I think people have got used to communicating online. Lots of friends of mine have played bridge online*,* and still doing so even though Covid is largely behind them.’ Interview participant ID10*.

The yoga teachers and participants were equally supportive of promoting online GYY classes to the public, seeing potential for the provision of classes within both community- and NHS-based settings. Technology-related access barriers were considered remediable, with the caveat of internet challenges in rural and remote communities, and pre-class training on internet security and Zoom usability would enhance confidence and engagement. They further suggested that as the benefits of online delivery were independent of age, the target demographic of GYY should be broadened from older adults to include anyone with health, mobility, or caregiver concerns impacting their ability to leave the home:

*‘I think it’s a whole new world. And I think technology can bring more people together and make people feel part of something else. You know*,* it is definitely another string to the bow to keep the National Health looking after the welfare of people’. Interview participant ID4*.

Participants also raised implementation concerns in the wider community, for older adults who may not have financial access to the internet or a suitable device for streaming online classes, or the digital literacy required for online engagement. Teacher solutions included setting up an equipment loan scheme modelled on existing services. Community-based infrastructures such as internet cafes could provide digital learning in a social environment, whereby students are matched with older adults to help them develop their digital competency.

## Discussion

Covid-19 social restrictions necessitated rapid transition of the GYY trial intervention from pre-planned face-to-face classes to online Zoom classes. A process evaluation involving class observations and interviews indicated both participants and yoga teachers viewed the online classes as a safe, acceptable, and sometimes preferable option for older adults with multimorbidity. Interlinking features of accessibility, technology, teaching adaptations, and safety collectively outweighed barriers associated with restricted real-time communication and digital literacy. While online classes in a range of contexts were instigated as a solution to Covid-19 restrictions, they have emerged as a standard option for the future health management of multimorbid older adults.

The trial findings support and extend the evidence base for online delivery of exercise and mind-body activities in both older adult [45–47] and clinical populations [22, 23, 25, 48]. A recent trial of yoga for falls prevention in adults over 60 years found a mid-intervention conversion to online delivery retained the value, health benefits, and social engagement of the original face-to-face classes, with some participants valuing the online format over face-to-face engagement [37]. Encouragingly, the GYY online classes saw continued recruitment in the older old, aged 80 years and older, a demographic that previously declined following conversion from face-to-face to online yoga delivery in another study [48].

### Accessibility

The primary benefit of GYY online delivery was improved accessibility. A picture emerged of these older adults as a proactive and engaged cohort, whose busy lives required new routines to ‘fit’ into their pre-existing schedules. For many participants accessibility was a proxy for convenience, and directly attributed to their preference for online rather than face-to-face classes post-trial. Confirming previous research, participants noted the home-based online classes removed attendance, temporal, and financial barriers related to impaired mobility, acute health issues, carer responsibilities, lifestyle commitments, transport, class availability, and adverse weather [25, 37, 47]. These factors were interlinked; not having to drive to class saved fuel and parking costs, limited their time commitment to the duration of the class, and enabled consistency of practice when feeling too unwell to leave home. Noted counterpoints to accessibility, common to previous older adult tele-exercise interventions [27, 45–47] were environmental, financial, and digital exclusion. It has been suggested that tele-exercise may work better for those able to access a sufficiently sized, quiet practice space and computer equipment such as cameras [37, 45]; however, these pragmatics may be unachievable for those with limited financial resources.

Online group-based classes offered an enhanced learning environment for many participants. They valued the privacy and focus of Zoom, whereby having the option to view only the teacher removed the distractions of a face-to-face group setting. Previous research indicated privacy was a main factor in choosing online over in-person yoga among a cancer cohort, for those self-conscious about body image or inexperienced in yoga [25]. Our findings support and further this, indicating privacy was particularly valued by males and participants with anxiety who felt uncomfortable learning a new activity in view of others. Interestingly, the GYY teachers had not considered this aspect of the online classes. By confirming privacy as an engagement factor, we recommend tele-yoga providers educate attendees on screen view options, enabling those who value the socialisation of a group to practice in gallery view, and those who prefer privacy to practice in speaker view.

### Digital literacy and technology use

Our trial identified three key knowledge processes for optimising tele-yoga access: one-step Zoom access via an embedded email link, basic pre-class training on audiovisual controls including volume and mute control and screen view options, and clear, simple instruction.

Importantly, as some of the trial teachers were as new to the Zoom platform as the participants, there was an element of shared learning in the 12-week programme. This suggests the digital literacy needed for telehealth interventions may be related to a gap in knowledge rather than age [49]. Future resolution of this could involve the integration of IT staff into trial delivery teams and providing pre-programme and ongoing training and technical support to those both delivering and receiving online interventions [45, 46].

The independence older adults gain through learning digital skills has been compared with that of learning to drive [50]. Our findings support the view that older adults are not a homogenous group regarding digital literacy [49], with many digitally fluent participants challenging the ageist stereotype of older adults and technology [29]. For those less digitally literate, the GYY trial presented a unique opportunity to learn about online communication platforms in a supportive environment, confirming previous research of increased and more diverse internet use among older adults during the Covid-19 pandemic [28, 31].

Our study further confirmed that older adults’ familiarity and competence with everyday technology such as smartphones and emails is not by default transferable to the use of online communication platforms [45]. The additional knowledge requirement of audiovisual settings and passcode entry to access and optimise Zoom use proved complicated for some participants. However, they were motivated to (more fully) engage in an increasingly digital society [49] and embraced the opportunity to learn and use this technology when provided access to the training and equipment to do so. Previous research suggests this digital training be inclusive of the potentially low IT literacy of older adults and address security and privacy concerns [49, 50].

### IT issues

Technology-related challenges associated with the online yoga classes mirrored those from previous telehealth inventions in older adult and clinical cohorts [22, 24, 45–47]. While at times frustrating, both participant and teacher feedback supported evidence that the response cost of overcoming technology challenges was outweighed by the resultant benefits of home-based class engagement [45, 49]. This study further shows that many IT-related issues were common to those both delivering and receiving the online classes [37]. The addition of the pre-class one-to-one sessions were invaluable in addressing and resolving common issues associated with accessing equipment and optimising on-screen visibility prior to the first group class, and are recommended for future online programmes to promote uptake and engagement. However, addressing technical issues such as weak internet connectivity were beyond the remit of this trial.

### Safety

Tele-yoga is not without caution. While evidence suggests yoga is as safe as other exercise-based interventions [51], recent studies reported both teacher safety concerns regarding online yoga [46], and an increase in the severity and probability of intervention-related adverse events in online compared to face-to-face yoga participants [48]. Conversely, the GYY teachers had no online safety concerns beyond those inherent to face-to-face yoga classes, reflecting trial recording of no serious or unexpected adverse events related to the intervention [32]. For the teachers, concerns regarding restricted participant visibility were offset by training, reduced class size and provision of large screen monitors.

For participants, the key aspect to online safety was ‘see and be seen’, whereby teachers offset the lack of real-time communication through their teaching style. Consistent with older adult cohorts, confirmation of their visibility could be as simple as teachers using their names [37, 46] and adopting a proactive teaching approach centred on simple verbal instruction, multi-angle demonstration of postures, and participant nonverbal feedback [37].

### Future implementation

Despite the increasing prevalence of multimorbidity and social isolation, and their negative health impact [52], access to rehabilitation programmes for some LTHCs is very limited [53]. With retirement age increasing beyond 65 years, and technology use common throughout a working life, age-related digital exclusion is anticipated to reduce over time [30]. This, combined with evidence for the biopsychosocial benefits of yoga (e.g [16–18, 20]), and these trial findings present online GYY classes as a safe, acceptable, and viable health management option.

Digital exclusion can be addressed on multiple levels, including social support, social motivation, and home-based learning [28, 30, 31]. Firstly, family and friends need to be recognised as an accessible support network. Providing knowledge, support, and computer equipment, their input addresses financial and literacy barriers to online engagement. Secondly, community-based training utilising existing voluntary and community sector (VCS) and public library services (e.g [50]) could enhance digital literacy skills, with the potential for remote training [28]. Thirdly, health and VCS organisations could be engaged to develop an equipment loan scheme, addressing barriers of lack of equipment (e.g [50]). Finally, and recognising engagement as a means of addressing social isolation [28, 49], online forums could be integrated into tele-health programmes to enable attendees to engage with others outside class [25].

Marketing of online yoga should emphasise both physical and technical accessibility. As well as providing access for those with health or transport barriers, marketing campaigns should highlight that joining a Zoom class is as simple as accessing an email message. Suggested marketing streams should target both digital users and non-users. Advertisements could be placed on social media platforms favoured by older adults, such as Facebook and YouTube, together with audiovisual or paper-based marketing at GP surgeries, libraries, and supermarket-based community notice boards.

### Strengths and limitations

These findings are grounded in robust methodology. Observations, field notes, and iterative analysis supplemented and informed interview data collection, and longitudinal data enabled investigation of participants preferences for and engagement in online delivery options beyond the context of the trial period. Bias was minimised by gathering teacher and participant feedback across all study sites, with the benefit of several teachers in the pilot phase of the trial having experienced both face-to-face and online delivery of the yoga programme. While qualitative data is limited in generalisation, this study’s breadth of sampling and data sources has enabled both the confirmation of previous smaller qualitative studies, and further added to the evidence base in the fields of tele-yoga for older adult and clinical populations.

Limitations to the sample size were minimised by purposeful sampling across confounding demographics including age, gender, socioeconomic indicators, and number and severity of LTHCs. However, it is noted that an inability to purposely sample based on ethnicity, due to the predominantly White British population in the main trial, limits generalisability of these findings to a broader range of ethnic groups due to lack of representation.

As participants needed a basic degree of computer literacy and in situ equipment to be eligible for the trial, and hence for this qualitative evaluation, there is an inherent bias to our findings in that they only represent the views of those with both internet access and an interest to engage in online classes. As such, they may not represent the views of those unable or disinterested to engage, and these views would be of interest to collect in future research. However, participant and teacher feedback has provided valuable information on how to address potential access barriers related to lack of computer or internet access, improve engagement, and promote interest and inclusivity going forward among older adult and clinical cohorts.

## Conclusion

Chair-based yoga classes within the GYY trial were rapidly transitioned from face-to-face to online Zoom-based delivery in response to Covid-19 social gathering restrictions. Both participants and yoga teachers viewed these online classes as a safe, acceptable, and sometimes preferable option for older adults with multimorbidity, supporting the role of tele-yoga for health management in the UK. The primary benefit of online delivery was improved accessibility for those experiencing difficulty leaving the home or engaging in group activities due to impaired mobility, acute health issues, carer responsibilities, busy lifestyles, transport issues, or adverse weather. Online delivery also removed geographical barriers associated with lack of local classes and enabled participants to continue classes with a preferred teacher post-trial. Technical issues were mostly resolvable, with minimal digital literacy required to access the Zoom classes, and teaching adaptations minimising communication restrictions inherent to online delivery. Based on this, participants and teachers suggested online delivery of GYY classes was suitable for a wider demographic than the trial cohort of older adults with multimorbidity, and should be more broadly offered to individuals for whom accessibility issues precluded participation independent of age. Going forward, establishing connections with existing health, voluntary, and community-based organisations presents a potential pathway for addressing financial and digital barriers of equipment and internet access to online classes, through the development of equipment loan schemes and community-based training.

## Electronic supplementary material

Below is the link to the electronic supplementary material.

Supplementary Material 1

## Data Availability

All relevant data are included in the manuscript.

## Abbreviations

GYY: Gentle Years Yoga
IT: Information technology
LTHC: Long term health condition
VCS: Voluntary and community sector
