# Supplementary material for: Acceptability and feasibility of online delivery of chair-based yoga for older adults with multimorbidity – lessons from a process evaluation of the gentle years yoga trial
Source: BMC Complement Med Ther. 2025 Mar 17;25:107. doi: 10.1186/s12906-025-04838-6 (PMC11912673; doi:10.1186/s12906-025-04838-6)
Supplement: Supplementary file 1 — Supplementary Material 1 [file 12906_2025_4838_MOESM1_ESM.docx]

Acceptability and feasibility of online delivery of chair-based yoga for older adults with multimorbidity – Lessons from a process evaluation of the Gentle Years Yoga trial.

Supplementary file 1. Semi-structured interview schedule developed specifically for the Gentle Years Yoga online delivery study.

1. How did you find the one-to-one session before the first group class? (Prompt for if it was helpful, and why/why not).
2. What has been your experience of the online yoga classes? (Prompt for what did or didn’t work well).
3. How have you found the ability to communicate and engage with others online, compared to other classes or activities where you are in the same room with each other? (Prompt for what techniques they use and/or found useful).
4. How have you set up the home environment to take part in the online classes? (Prompt for any practical problems).
5. During the class observations I noted that some participants are not in full body view. How do you feel about this in terms of safety?
6. How do you feel about the safety of online yoga classes?
7. What, if any, IT issues have you experienced during the online classes? (Prompt for how confident they are with dealing with these).
8. How confident do you feel using Zoom? (Prompt for experience of using online delivery platforms, and the process of accessing the Zoom classes via the email link).
9. What are your thoughts on engaging in online yoga or other activities in the future after your experience of the yoga trial? (Prompt for accessibility factors of home-based online classes compared to community-based face-to-face classes)
10. Is there anything that we haven’t covered in the interview that you would like to talk about or that you suggest we should know?
